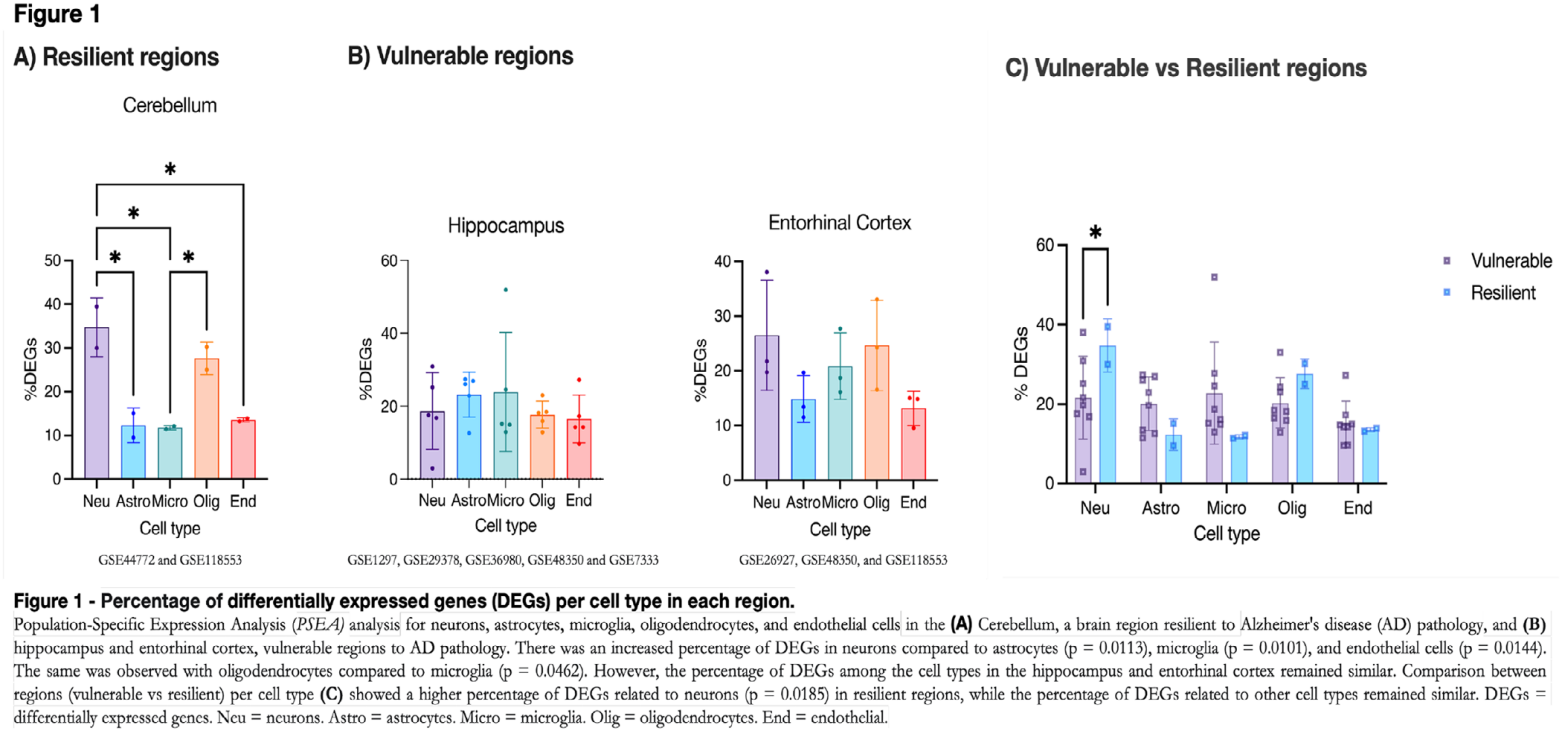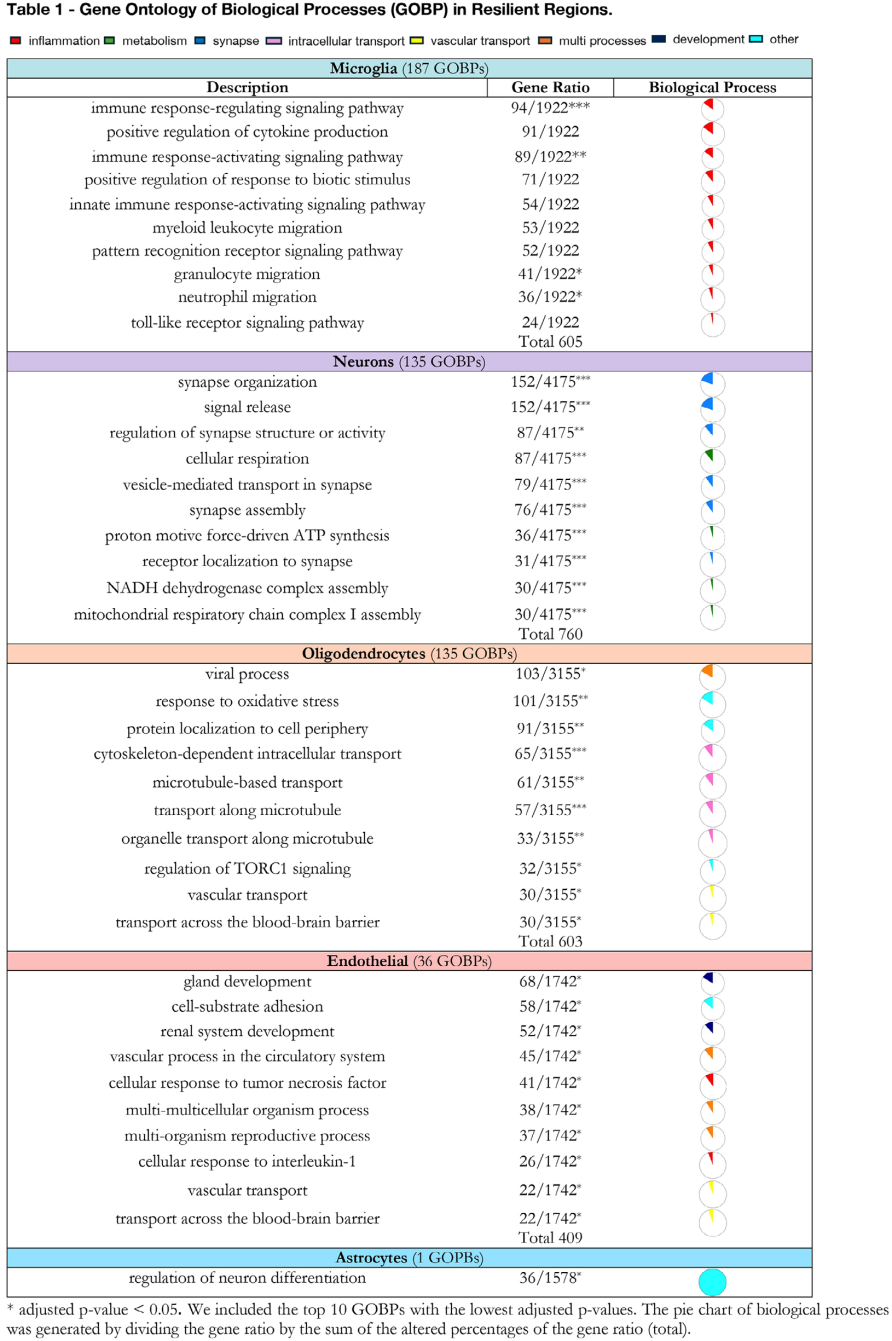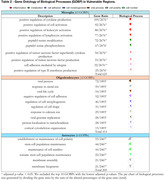# Brain cell transcriptomics in vulnerable and resilient regions in Alzheimer’s Disease brains

**DOI:** 10.1002/alz.091719

**Published:** 2025-01-09

**Authors:** Gabriela Mantovani Baldasso, Giovanna Carello‐Collar, Christian Limberger, Rodrigo Sebben Paes, Marco De Bastiani, Eduardo R. Zimmer

**Affiliations:** ^1^ Universidade Federal do Rio Grande do Sul, Porto Alegre, Rio Grande do Sul Brazil; ^2^ Universidade Federal do Rio Grande do Sul, Porto Alegre, RS Brazil; ^3^ Universidade Federal do Rio Grande do Sul, Porto Alegre Brazil; ^4^ Brain Institute of Rio Grande Do Sul, PUCRS, Porto Alegre, RS Brazil; ^5^ McGill Centre for Studies in Aging, Montreal, QC Canada

## Abstract

**Background:**

Neurodegeneration is a major pathological feature of Alzheimer’s disease (AD). During this process, it is known that not only neurons are affected but also glial cells. However, the biological mechanisms driving brain cellular vulnerability and resilience to neurodegeneration in AD remain elusive. Thus, we aimed to investigate the transcriptomic profile of brain cell types in vulnerable and resilient AD regions.

**Method:**

We searched microarray AD datasets in the Gene Expression Omnibus repository for available transcriptomics of vulnerable (hippocampus and entorhinal cortex) and resilient (cerebellum) postmortem regions. The data was downloaded using the GEOquery package. We performed gene expression deconvolution with the Population‐Specific Expression Analysis (PSEA) to obtain the differentially expressed genes (DEGs) specific for neurons, astrocytes, microglia, oligodendrocytes, and endothelial cells (FDR‐adjusted p‐value < 0.05). We then executed functional enrichment analysis to obtain Gene Ontology (GO) of Biological Processes (GOBP) terms (clusterProfiler package). All analyses were done in R.

**Result:**

We included eight GSEs to vulnerable regions, comprising 165 cognitively unimpaired (CU) and 182 AD individuals, and two for resilient (123 CU, 179 AD). The PSEA analysis revealed an increased percentage of DEGs in neurons and oligodendrocytes in resilient regions when compared with astrocytes, microglia, and endothelial cells (Figure 1A). By contrast, the percentage of DEGs among the cell types in vulnerable regions remained similar (Figure 1B). Interestingly, resilient regions presented more DEGs related to neurons than the vulnerable ones (Figure 1C). Microglia were the cell type with the most GOBPs number in resilient regions, while astrocytes had one (Table 1). Table 2 shows that only microglia, oligodendrocytes, and astrocytes presented GOBPs in the vulnerable regions.

**Conclusion:**

We demonstrate that neurons are the only cell type presenting a higher number of DEGs in resilient regions compared to vulnerable regions. Other cells exhibit a similar percentage of DEGs in both resilient and vulnerable regions. Regarding GOBPs, all cell types show enriched terms in resilient regions; however, in vulnerable regions, neurons and endothelial cells do not exhibit any enriched terms. These findings suggest that neurons possess an exceptional ability to adapt in resilient regions, a trait not observed in vulnerable regions.